# Complement system biomarkers in first episode psychosis

**DOI:** 10.1016/j.schres.2017.12.012

**Published:** 2019-02

**Authors:** Maja Kopczynska, Wioleta Zelek, Samuel Touchard, Fiona Gaughran, Marta Di Forti, Valeria Mondelli, Robin Murray, Michael C. O'Donovan, B. Paul Morgan

**Affiliations:** a Systems Immunity University Research Institute, Cardiff University, Cardiff CF144XN, UK; b MRC Centre for Neuropsychiatric Genetics and Genomics, School of Medicine, Cardiff University, Cardiff CF144XN, UK; c Institute of Psychiatry, Psychology and Neuroscience, King's College, De Crespigny Park, London SE5 8AF, UK

**Keywords:** Complement, Inflammation, Biomarkers, Predictors

## Abstract

Several lines of evidence implicate immunological/inflammatory factors in development of schizophrenia. Complement is a key driver of inflammation, and complement dysregulation causes pathology in many diseases. Here we explored whether complement dysregulation occurred in first episode psychosis (FEP) and whether this provides a source of biomarkers. Eleven complement analytes (C1q, C3, C4, C5, factor B [FB], terminal complement complex [TCC], factor H [FH], FH-related proteins [FHR125], Properdin, C1 inhibitor [C1inh], soluble complement receptor 1 [CR1]) plus C-reactive protein (CRP) were measured in serum from 136 first episode psychosis (FEP) cases and 42 mentally healthy controls using established in-house or commercial ELISA. The relationship between caseness and variables (analytes measured, sex, age, ethnicity, tobacco/cannabis smoking) was tested by multivariate logistic regression.

When measured individually, only TCC was significantly different between FEP and controls (p = 0.01). Stepwise selection demonstrated interdependence between some variables and revealed other variables that significantly and independently contributed to distinguishing cases and controls. The final model included demographics (sex, ethnicity, age, tobacco smoking) and a subset of analytes (C3, C4, C5, TCC, C1inh, FHR125, CR1). A receiver operating curve analysis combining these variables yielded an area under the curve of 0.79 for differentiating FEP from controls. This model was confirmed by multiple replications using randomly selected sample subsets.

The data suggest that complement dysregulation occurs in FEP, supporting an underlying immune/inflammatory component to the disorder. Classification of FEP cases according to biological variables rather than symptoms would help stratify cases to identify those that might most benefit from therapeutic modification of the inflammatory response.

## 1 Introduction

First episode psychosis (FEP) refers to patients who have presented with and received treatment for their first psychotic episode (Weiden et al., 2007), which may be the first presentation of disorders such as schizophrenia, schizoaffective disorder or bipolar disorder. The aetiology and pathogenesis of psychosis is not yet fully understood, although the weight of evidence suggests a contribution from both genetic and environmental factors (Tsuang et al., 2004, Brown, 2011). In the absence of a biological marker, current diagnosis relies upon clinical assessment, and treatment is largely empirical (Li et al., 2012). It is therefore not surprising that treatment response rates are unsatisfactory; multiple treatment failures are common and relapse is frequent (Emsley et al., 2013). The lack of biomarkers also contributes to difficulties in early diagnosis, disease stratification, therapeutic choice and prediction of outcome (Weickert et al., 2013). A handful of plasma and serum biomarkers have been described but have often failed to replicate, have not gained widespread acceptance and have not provided significant insight into pathogenesis or optimal therapeutic strategy (Weickert et al., 2013). The goal of the current study was to investigate the potential for using complement-related proteins as serum biomarkers with prognostic and/or theranostic value.

A potential link between inflammation and both FEP and schizophrenia has been proposed for decades (Ganguli et al., 1987, Kirch, 1993, Goldsmith et al., 2016); however, the usefulness of inflammatory or immunological markers is yet to be established. Several lines of evidence, particularly in schizophrenia, suggest that immunological factors contribute to the development of psychosis (van Kesteren et al., 2017). Maternal virus infection has been proposed as a risk factor for schizophrenia and bipolar disorder (Schmitt et al., 2014, Parboosing et al., 2013), and inflammatory cytokines such as interleukin (IL)-1, IL-6 (Drzyzga et al., 2006) and transforming growth factor (TGF)-α (Kim et al., 2009), which drive inflammation and act as modulators of immune response, are increased in plasma in some schizophrenia patients (Watanabe et al., 2010). Importantly, treatment with anti-inflammatory medication is associated in some studies with reduction of core symptoms of schizophrenia (Drzyzga et al., 2006). The data on immunological/inflammatory factors is, however, inconsistent, suggesting that a targeted approach focusing on specific inflammatory pathways might be more rewarding.

The complement system is a major effector of innate immunity and an adjuvant of adaptive immunity. Complement comprises around 30 plasma and cell-surface proteins that interact with one another to induce a series of inflammatory responses involved in defence against infection (Ricklin et al., 2010). Complement is activated via three initiating pathways (classical, alternative and lectin), all of which converge on a common effector pathway with formation of the membrane attack complex (MAC). Complement acts through production of: 1. opsonins (C3b, iC3b) increasing the ability of macrophages and neutrophils to phagocytose; 2. anaphylatoxins (C3a, C5a) inducing local and systemic inflammatory responses, increasing the permeability of blood vessels and attracting neutrophils; 3. through direct killing of organisms by formation of the MAC which disrupts the phospholipid bilayer of a target cell (Ingram et al., 2012). Complement system function is regulated (accelerated or inhibited) by complement regulatory proteins present in fluid-phase or membrane-bound (Noris and Remuzzi, 2013).

Dysregulation of the complement system in schizophrenia has been noted in a few small studies, notably increased activation of the classical complement pathway (Mayilyan et al., 2008, Hakobyan et al., 2005), and decreased complement hemolytic activity, indicative of consumption (Mayilyan et al., 2008). However, data regarding complement changes are inconsistent, with one study reporting increased plasma levels of C3 and C4 (Mayilyan et al., 2008), while another described decreased levels of these proteins (Wong et al., 1996). This lack of consensus, together with a growing evidence base indicating that complement is involved in brain development (Stephan et al., 2012, Stevens et al., 2007), as well as the recent genetic study implicating *C4* as a susceptibility locus for schizophrenia (Sekar et al., 2016), has motivated us to explore whether changes in the complement system can be detected in FEP in comparison with healthy controls. Here we describe the measurement in serum from FEP cases and healthy controls of a selected panel of complement proteins and activation products with the aim of gaining insight into underlying pathology and identifying candidate biomarkers. The marker set was chosen to interrogate classical (C1q, iC3b, C3, C4), alternative (Properdin, FB, FH, iC3b) and terminal (TCC) activation pathways. Selected markers have been implicated in pathophysiology of mood disorders such as schizophrenia (Hakobyan et al., 2005, Mayilyan et al., 2008) and bipolar affective disorders (Akcan et al., 2017), and/or neurological and neurodegenerative disorders such as Alzheimer's disease (Kolev et al., 2009), Parkinson's disease (Loeffler et al., 2006), multiple sclerosis (Ingram et al., 2012) and epilepsy (Aronica et al., 2007, Jamali et al., 2010). CPR was measured because of its widely accepted use as a benchmark of inflammatory state.

## 2 Methods

### 2.1 Samples

In the present study, 136 FEP patients and 42 mentally healthy controls were recruited as part of the Physical health and substance Use Measures in Psychosis (PUMP) and the Genetics and Psychosis (GAP) studies. The demographics of all study participants is presented in Table 1. All patients aged 18–65 years who presented with FEP were approached. The age of onset of psychosis was within 6 months of presentation. Patients met the ICD-10 criteria for psychosis (codes F20–29 and F30–33) and were recruited from mental health Trusts in London and South-East England. Patients were interviewed using the Schedules for Clinical assessment in Neuropsychiatry (SCAN) present state examination protocol and ICD-10 diagnoses were derived from the Operational Criteria (OPCRIT) algorithm (Rucker et al., 2011). If patients were too unwell to cooperate, they were re-contacted after the start of treatment. The majority of the patients were not drug naïve at the time of blood sample collection; details of drug therapy were not available for the majority. Volunteer controls, recruited using internet and newspaper advertisements and by distributing leaflets at train stations, shops, and job centres, were administered the Psychosis Screening Questionnaire (Bebbington and Nayani, 1995) and were excluded if they met the criteria for a psychotic disorder or if they reported a previous diagnosis of psychotic illness. Ethnicity was self-reported. Some ethnicity groups were merged to provide bigger populations for statistical analysis. The two smallest groups remaining (Mixed and Asian) could not easily be merged with another ethnicity group. The original number of controls was low compared to the cases and was further reduced by removing from analysis samples of mothers of cases previously used as controls in the GAP study. All cases and controls included in the study gave written informed consent. Ethical approval was granted by the Research Ethics Committee of The Joint South London and Maudsley and The Institute of Psychiatry NHS Research Ethics Committee.

**Table 1:** The demographics and lifestyle variables of the study population. χ^2^; chi-square test, W; Mann-Whitney test. Variables significant at p = 0.05 are in bold and underlined.

Variable	Controls n (%)	Cases n (%)	Test statistic	p-Value
**Numbers**	**42 (100%)**	**136 (100%)**		
Age in years, mean (SD)	37.55 (14.44)	32.51 (10.12)	W = 3343.5	0.09
Sex, male	19 (45.24%)	96 (71.11%)	χ^2^ = 7.94	**0.005**
Tobacco smoking	12 (28.57%)	100 (73.53%)	χ^2^ = 15.33	**0.001**
Cannabis smoking	14 (33.33%)	94 (69.12%)	χ^2^ = 4.11	**0.04**
Tobacco and cannabis smoking	9 (21.43%)	83 (61.03%)	χ^2^ = 10.29	**0.001**
Ethnicity				
White British	20 (47.62%)	38 (27.94%)	χ^2^ = 4.80	**0.03**
Mixed	3 (7.14%)	5 (3.68%)	χ^2^ = 0.27	0.60
White other	5 (11.90%)	17 (12.50%)	χ^2^ = 0	1
Various Asian	4 (9.52%)	8 (5.88%)	χ^2^ = 0.22	0.64
Black Caribbean	3 (7.14%)	26 (19.12%)	χ^2^ = 2.55	0.11
Black African	3 (7.14%)	28 (20.59%)	χ^2^ = 3.15	0.08
No information	4 (9.52%)	14 (10.29%)	χ^2^ = 0	1

### 2.2 Immunoassays

The concentrations of 11 complement analytes: C1q, C3, C4, C5, Factor B (FB), Factor H (FH), FH-related proteins 1, 2 and 5 (FHR125), Properdin, C1 inhibitor (C1inh), soluble complement receptor 1 (sCR1), and terminal complement complex (TCC) were measured using established in-house enzyme-linked immunosorbent assays (ELISA) (Table 2). C-reactive protein (CRP) was measured using a commercial ELISA (CRP Duoset DY1707; R&D Systems, Abingdon, UK). The samples available for analysis comprised serum aliquots (0.2–0.5 ml) that had been stored at − 80 °C and not subjected to freeze-thaw. For in-house ELISA, Maxisorp (Nunc, Loughborough, UK) plates were coated with affinity-purified capture antibody overnight at 4 °C, and blocked (1 h at room temperature (RT)) with either 3% non-fat dried milk (NFM) or 1% bovine serum albumin (BSA) in phosphate-buffered saline containing 0.1% Tween 20 (Sigma Aldrich) (PBS-T). After washing wells in PBS-T, purified protein standards or serum samples optimally diluted in 1% NFM or 0.1% BSA in PBS-T, were added in duplicate and incubated for 1.5 h at 37 °C. Different sample dilutions were used for different assays (Table 2). Wells were washed 3 × with PBS-T then incubated (1 h) at RT with detection antibody (unlabelled or labelled with horseradish peroxidase (HRP)) and washed 3 × with PBS-T. For assays using unlabelled detection antibodies, HRP labelled secondary antibody (anti-mouse or anti-rabbit IgG as appropriate) was added to wells, incubated and washed as above. Signals were detected using o-Phenylenediamine dihydrochloride (OPD, SIGMAFAST™, Sigma-Aldrich) and absorbance (492 nm) was measured. Standards were included on each plate and samples from controls and cases were randomly assigned to eliminate assay bias. A nonlinear regression model was used to fit standard curves generated by ELISA. Total protein concentration (μg/ml) was automatically calculated by reference to the standard curve using GraphPad Prism. Detection limits, working ranges and assay performance were determined as described (Ingram et al., 2012), using serum from 62 local healthy controls.

**Table 2:** The table lists the antibody pairs used in the multiplex sets, the sources of the antibodies and the standards, dilutions and assay working range. MM; mouse monoclonal antibody, RP; rabbit polyclonal antibody, HRP; horseradish peroxidase (antibodies labelled in-house), TCC; terminal complement complex; C1Inh; C1 inhibitor; FB; Factor B; FH; Factor H; FHR125; Factor H related proteins 1, 2, and 5; CR1; complement receptor 1; CRP; C-reactive protein. A kind gift from SRdC – Prof S. Rodriguez de Cordoba, Madrid. ECACC: European Collection of Authenticated Cell Cultures, Hycult: http://www.hycultbiotech.com/; CompTech: http://www.complementtech.com/; R&D Systems: http://www.rndsystems.com/.

Assay	Capture antibody	Detection antibody	Standard	Working range (ng/ml)	Sample dilution
C1q	MM anti-C1q mAb (WL02, Hycult)	MM anti-C1q (DJ01, Hycult)-HRP	C1q (in-house purified)	32–1000	1:1000
C3	RP anti human C3 (in-house)	RP anti-C3 (in-house)-HRP	C3 (CompTech)	32–1000	1:16,000
C4	RP anti-C4 (in-house)	RP anti-C4 (in-house)-HRP	C4 (CompTech)	8–500	1:4000
C5	RP anti-C5 (in-house)	MM anti-C5 (2D5; in-house)-HRP	C5 (in-house purified)	32–1000	1:200
Factor B	MM anti-FB (JC1; in house)	MM anti-FB (MBI-5; in-house)-HRP	FB (in-house purified)	64–1000	1:500
Factor H	MM anti-FH (OX24; ECACC)	MM anti-FH (35H9; in-house)-HRP	FH (in-house purified)	16–1000	1:3000
C1inh	MM anti-C1inh (in-house)	RP anti-C1inh (in-house)-HRP	C1 inhibitor (Cinryze drug)	4–100	1:16,000
Properdin	MM anti-properdin (1.1.1; Gift of SRdC)	MM anti-properdin (12-14-2; gift of SRdC)-HRP	Properdin (CompTech)	7–100	1:400
FHR125	MM anti-FHR125 (MBI125; in-house)	RP anti-FH (in-house)-HRP	FHR125 (in-house)	4–250	1:4000
TCC	MM anti-TCC (aE11, Hycult)	MM anti C8 (E2, in-house)-HRP	TCC (in-house purified)	60–1000	1:50
CR1	RP anti-CR1 (in-house)	MM anti-CR1 (MBI35; in-house)-HRP	CR1 (in-house purified)	16–500	1:2
CRP	MM anti-CRP (R&D systems; DuoSet)	MM anti-CRP-biotin/avidin-HRP (R&D systems; DuoSet)	CRP (R&D systems; DuoSet)	0.8–50	1:200

### 2.3 Statistical methods

Univariate statistical tests were performed using the Mann-Whitney *U* test for comparison between the cases and controls, for each analyte. Univariate associations between the condition and categorical variables such as sex or ethnicity were tested using the Chi-square test. In order to avoid loss of information and maximise the power of the study, we used all the samples and variables that were available. Stepwise logistic regression models were tested, including the measured analytes together with co-variates such as sex, age, ethnicity and the consumption of tobacco and cannabis to adjust for their impact on measured analytes in the cohort. Due to the relatively small number of samples available, we chose to perform stepwise selection on the complete available dataset. The selected models were then tested using Receiver Operating Curve (ROC) analyses, with leave-one-out cross-validation. In order to rule out the possibility of over-fitting of the model using this approach, we performed replications of stepwise models on different subsets of the data. The most stable variables - those most often selected and most significant - were identified. For each replicate, an AUC was computed and the distribution of AUCs across the replicates measured. All tests and analyses were performed with the statistical software R, including the pROC packages.

## 3 Results

### 3.1 Serum levels of TCC and demographic variables distinguish FEP from control

Of twelve analytes measured, only one, TCC, was significantly different between FEP and control populations (controls, 36.22 μg/ml; FEP 30.29 μg/ml: p = 0.01 Table 3). C1q levels were lower in FEP patient serum compared to controls at a trend level (controls, 134.70 μg/ml; FEP, 128.31 μg/ml: p = 0.07). Application of the Bonferroni correction resulted in a p-value for TCC of 0.12, indicating only a trend but not significance; however, this is a very conservative test, particularly in the presence of many dependent variables, because the Bonferroni test assumes the multiple tests to be independent. A robust, and computationally more intensive, alternative to the Bonferroni correction is the permutation correction (Sham and Purcell, 2014); this correction gave an adjusted p-value for TCC of 0.014. The association of FEP with sex, ethnicity, tobacco and cannabis consumption was assessed using Chi-square tests; all four of these categorical variables showed significant or near-significant effect (sex, p < 0.005; ethnicity, p < 0.07; tobacco, p < 0.00005; cannabis, p < 0.04). These differences in sex, ethnicity, tobacco and cannabis may have arisen, at least in part, as a consequence of the ascertainment protocols; we include them in the models below to adjust for any potential impacts on measured analytes. Of note, the majority of the patients were not drug naïve at the time of blood sample collection and details of drug therapy were not available; however, one of us recently reviewed the impact of psychotropic drugs on inflammation/immunity and found no consistency for reported effects in the literature (Baumeister et al., 2016).

**Table 3:** Complement analyte differences between first episode psychosis (FEP) and controls. Significance of differences was tested using the Mann-Whitney test. Variables significant at p = 0.05 are in bold and underlined. TCC; terminal complement complex; C1inh; C1 inhibitor; FB; Factor B; FH; Factor H; FHR125; Factor H related proteins 1, 2, and 5; CR1; complement receptor 1; CRP; C-reactive protein.

Assay	FEP		Control		p Value
	Mean (μg/ml)	StDev	Mean	StDev	
C1q	128.31	28.53	134.70	27.34	0.07
C3	1698.74	1973.34	1871.69	2119.61	0.46
C4	475.39	129.39	463.42	143.23	0.70
C5	81.37	38.60	91.56	45.23	0.30
FB	102.46	35.62	107.20	40.93	0.72
FH	383.18	185.39	357.89	136.71	0.76
C1inh	101.63	31.12	105.68	39.66	0.88
Properdin	4.34	2.85	4.46	2.69	0.76
FHR125	9.69	6.33	10.67	5.42	0.31
TCC	30.29	14.54	36.22	12.85	**0.01**
CR1	0.02	0.01	0.02	0.01	0.52
CRP	4.78	5.65	3.76	3.40	0.72

### 3.2 Logistic regression and development of prediction models

**Fig. 1:**
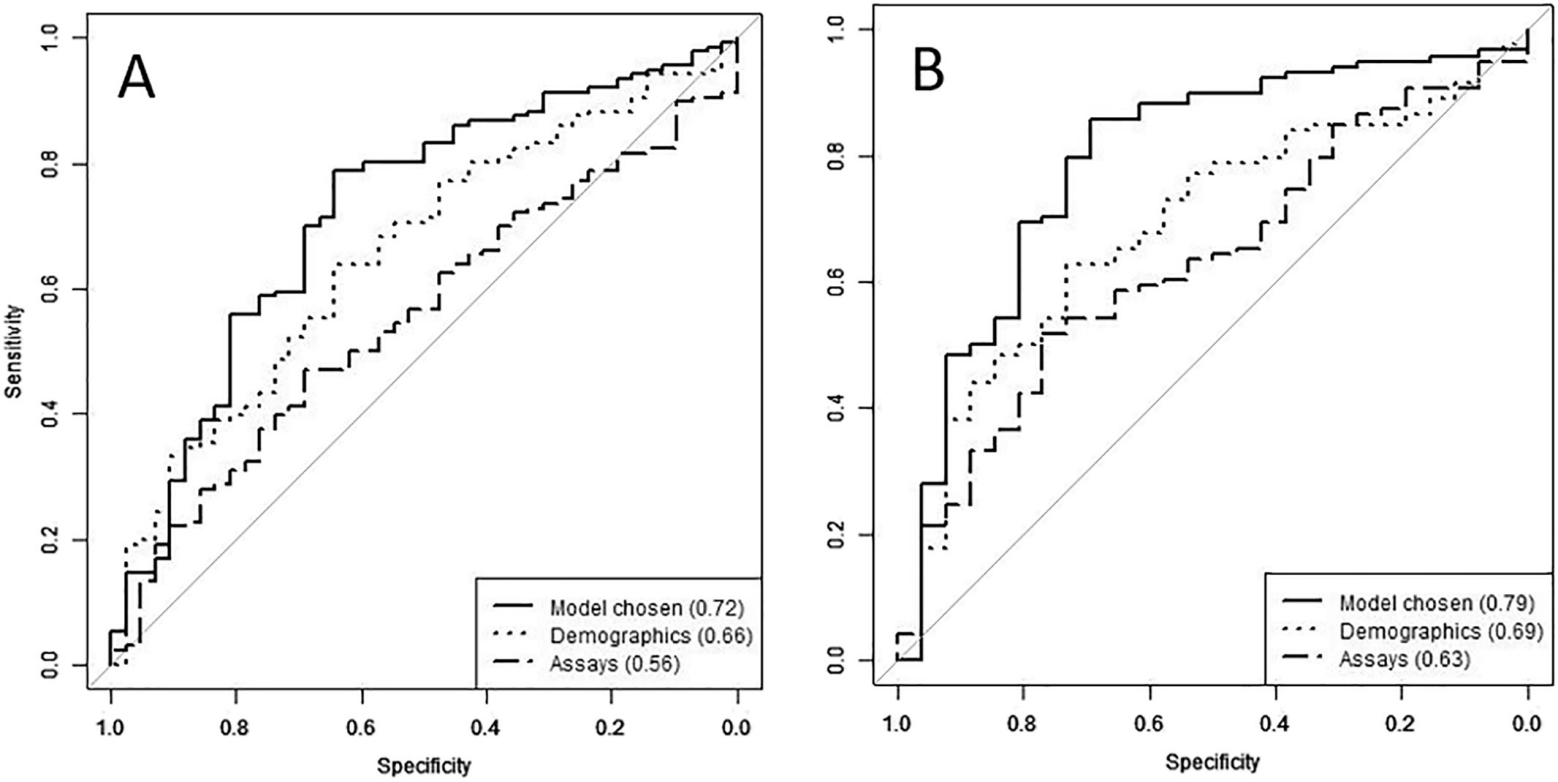
Receiver operated characteristic (ROC) curves to predict the probability of FEP compared to control subjects. The first model (A) comprised demographics (sex, age, ethnicity) and contributing analytes TCC and CRP; this gave an AUC statistic of 0.72. The values of sensitivity and specificity were respectively 0.79 and 0.64, with a minimised difference probability threshold (MDT) of 0.72.The second model (B) included tobacco smoking status as an additional demographic factor and contributing analytes comprised C3, C4, C5, TCC, C1inh, FHR125, CR1; this gave an AUC statistic of 0.79. This model has respective sensitivity and specificity of 0.85 and 0.69, with MDT = 0.78.

To find the set of analytes and demographics that best distinguished FEP and controls, different combinations were tested in multiple logistic regression models. We fitted a logistic regression to establish predictive values of complement risk factors. We then performed a stepwise procedure, to only include in the model the significant variables (details of the analyte selection are shown in Supplementary Table 1). The first multivariate model (Model A) was tested on the whole study population (178 samples: 136 FEP; 42 controls). Because data on smoking status was not available for a significant proportion (34/178) of the sample, this variable was omitted from the Model A. The resulting model after stepwise selection comprised sex, age, ethnicity, TCC and CRP (Table 4A). People of black Caribbean and black African ethnicities were significantly over-represented in the cases in comparison to control populations. With this selected model, a leave-one-out cross-validation was performed on the dataset to estimate its accuracy. At each iteration, one sample was removed, the model was trained on the remaining samples, coefficients were estimated, enabling the evaluation of the probability of caseness for the sample that had been removed. Predictions were then compared to the actual outcomes, resulting in an AUC of 0.72 (Fig. 1A). Sensitivity and specificity in the model depend on the prediction probability threshold which classifies the samples into two groups, “predicted cases” and “predicted controls”. The lower the threshold, the more cases are predicted and the more likely the actual cases are correctly predicted, thus sensitivity increases, and vice versa. The optimal threshold was obtained by minimising the difference between sensitivity and specificity; the values of sensitivity and specificity were respectively 0.79 and 0.64, with a minimised difference probability threshold (MDT) of 0.72.

**Table 4:** Stepwise selection of the logistic regression analysis including the demographics, (A) First model – Model A, tested on the whole study population without tobacco variable; (B) Second model – Model B tested on the study population with tobacco variable available. Variables significant at p = 0.05 are in bold and underlined. Ethnicity: White British was taken as a reference group. TCC – terminal complement complex; C1inh – C1 inhibitor; FB – Factor B; FH – Factor H; FHR125 – Factor H related proteins 1, 2, and 5; CR1 – complement receptor 1; CRP – C-reactive protein; OR – odds ratio; CI – confidence interval.

Variables	p-Value	logOR (95% CI)
A		
Sex (male)	0.051	0.81 (− 0.004; 1.62)
Age	**0.01**	− 0.04 (− 0.08; − 0.01)
Ethnicity: white British	n/a	n/a
Ethnicity: mixed	0.46	− 0.63 (− 2.31; 1.05)
Ethnicity: white other	0.15	0.92 (− 0.34; 2.17)
Ethnicity: various Asian	0.64	0.36 (− 1.14; 1.86)
Ethnicity: black Caribbean	**0.01**	1.81 (0.38; 3.24)
Ethnicity: black African	**0.004**	2.40 (0.76; 4.03)
Ethnicity: no information	0.24	0.84 (− 0.55; 2.23)
TCC	**0.001**	− 0.05 (− 0.08; − 0.02)
CRP	0.19	0.06 (− 0.03; 0.15)

B		
Sex (male)	**0.001**	2.55 (1.07; 4.03)
Ethnicity: white British	n/a	
Ethnicity: mixed	0.33	− 1.35 (− 4.05; 1.35)
Ethnicity: white other	0.97	− 0.03 (− 1.90; 1.84)
Ethnicity: various Asian	0.13	1.97 (− 0.57; 4.50)
Ethnicity: black Caribbean	**0.02**	2.98 (0.43; 5.53)
Ethnicity: black African	**0.01**	2.92 (0.78; 5.07)
Ethnicity: no information	0.11	1.98 (− 0.44; 4.40)
Tobacco	**0.0003**	2.81 (1.30; 4.33)
CR1	0.16	− 86.98 (− 207.87; 33.93)
TCC	0.098	− 0.04 (− 0.09; 0.01)
C3	**0.02**	− 0.0003 (− 0.0005; − 0.00005)
C4	**0.01**	− 0.009 (− 0.01; − 0.0024)
C5	0.09	− 0.01 (− 0.03; 0.002)
C1inh	0.08	− 0.02(− 0.03; 0.002)
FHR125	0.08	0.12 (− 0.02; 0.26)

According to multiple studies, tobacco and cannabis smoking are intimately associated with schizophrenia and could have implications for the underlying neurobiology of this illness (Kelly and McCreadie, 2000, de Leon et al., 2002, Andréasson et al., 1987, Moore et al., 2007). In our study, history of tobacco and cannabis smoking are both significantly over-represented in FEP patients in comparison to controls. Therefore, a second model (Model B) was tested to adjust for the smoking variables; missing data reduced the number of samples to n = 144. This second stepwise selection for the Model B comprised sex, ethnicity, tobacco smoking, and the complement analytes C3, C4, C5, TCC, C1inh, FHR125, CR1 (Table 4B). The most significant (p < 0.05) variables were sex, ethnicity, tobacco smoking, and the complement analytes C3 and C4. Male sex was over-represented in FEP cases in comparison to controls. Four additional complement biomarkers, C5, TCC, C1inh and FHR125, were significant at the 0.1 level and were included in the model. Of note, the age variable was not statistically significant (p = 0.55) in Model B. There was a weak but significant association between the age and the smoking status (p = 0.097) that mirrored the association between age and FEP status (young age associates with smoking associates with caseness). Because of the association with smoking status, included in Model B, the age variable no longer provided additional significance and was therefore omitted in Model B. We also observed weak but significant correlations between age and some of the complement factors included in Model B: C4 (Spearman correlation (sc) 0.21; p = 0.01), C5 (sc 0.16; p = 0.05) and FHR125 (sc 0.16; p = 0.05). CRP was not included in Model B; due to its correlation with tobacco smoking, it added no further value in the multivariate Model B. Cannabis consumption was not included in Model B; because of its strong association with tobacco smoking (p < 0.0001), adding this variable did not improve the model. The model was tested with leave-one-out cross-validation as described above; the final predictive value (AUC) was 0.79 (Fig. 1B). This second model has respective sensitivity and specificity of 0.85 and 0.69, with MDT = 0.78.

### 3.3 Analysis of correlations between variables and influence of potential confounding factors

Significant correlations between the complement analytes were identified; however, for most pairs the correlations were weak (Supplementary Fig. 1). These correlations were not considered in the final Models because their inclusion in tests did not improve the predictive performance; further, inclusion carries the risk of overfitting of the models.

We also investigated the influence of demographic and lifestyle variables on complement protein levels. Tobacco smoking was associated with increased levels of FH (p = 0.046) and reduced TCC (p = 0.008) (Supplementary Table 2). Cannabis consumption was associated with a significant reduction in C5 levels (p = 0.001) and trending reduction in C4 (p = 0.086), C1q (p = 0.051) and CR1 (p = 0.081) (Supplementary Table 3). Male sex was associated with lower TCC levels (p = 0.03) and higher C4 levels (p = 0.04) (Supplementary Table 4). In previous studies, a nonlinear association with age has been described for C3 and C4, which also differs in women and men (Ritchie et al., 2004); we found a positive association between age and C4 in women (p = 0.02), but not in men (p = 0.71) but no association between age and C3 in either sex.

### 3.4 Further analysis for incorrectly classified cases

We explored whether there were any specific differences between the correctly and incorrectly classified individuals in Model B. We identified 25 misclassified samples, 17 false negatives and 8 false positives from the 144 samples tested. Of these, 19 were female (13 false negatives, 6 false positives); Chi square testing gave p = 9.1e-6 for female sex as a factor in miss-classification. For tobacco consumption, 11 of the miss-classified samples were from non-smokers and 14 from smokers; Chi square testing gave p = 0.009 for smoking as a factor in miss-classification.

### 3.5 Sensitivity analysis

Black Caribbean and Black African ethnicities were much more frequent in cases compared to controls. To test the impact of this on the model, a stepwise model was fitted after removing these samples from both case and control sets. The resulting model comprised sex, ethnicity, tobacco consumption, C3, C4, C5, TCC and CRP. The predictive power of this model was comparable to that of Model B using the complete sample sets (AUC of 0.80). We also tested the impact of removing samples with CRP values above 10 μg/mL, because these might represent participants with acute infection. The resulting model, comprising sex, ethnicity, tobacco consumption, C3, C4, C5, TCC gave the same predictive power (AUC of 0.80).

### 3.6 Replication

In order to rule out the possibility of over-fitting of the model using this approach, we performed 100 replicates of stepwise models on different subsets of the data (containing all available analytes as demographics). For each replicate, the dataset was randomly split into a training set (80%) on which we performed a stepwise methodology, before testing the selected model on the remaining validation set (20%) and computing the corresponding AUC. The variables most strongly associated with FEP were sex, tobacco consumption and ethnicity, selected respectively 100, 96 and 79 times, and significant (p < 0.05) 92, 85 and 66 times (all out of 100 replications). After controlling for these co-variates, the most stable biomarkers were C4 and C3, included in the model 92 and 80 times, and significant (p < 0.05) 75 and 57 times. CR1, C5 and TCC were selected in the model > 50% of the time. These most stable co-variates and biomarkers were identical to those selected when performing stepwise regression on the whole dataset. The median AUC from these 100 replicates was 0.69 (standard deviation of 0.13). The decreased AUC compared to whole dataset analysis (0.79) can be explained by the smaller size of the training sets (115 versus 143).

## 4 Discussion

There has been debate over the past few years about the criteria currently used to classify patients with psychiatric disorders. The lack of objective and quantitative diagnostic criteria means that we rely on symptom-based categorisation when deciding on patient classification and management. A classification of patients according to underlying biology rather than symptoms would simplify the path towards effective and patient-appropriate treatments (Mondelli et al., 2015). Accumulating evidence has implicated inflammation and the immune system in the aetiology of psychosis (Najjar and Pearlman, 2015, Kodavali et al., 2014), suggesting that biological markers might emerge from a better understanding of these systems in psychiatry; complement is one such system (Hakobyan et al., 2005).

Complement has recently emerged as a key player in brain development; C1q and downstream classical pathway activation products mark synapses for elimination during post-natal brain remodelling, a process that is essential for brain development, maturation, and optimal function (Stevens et al., 2007). This physiological process has also been implicated in pathological synapse elimination in the context of schizophrenia and dementia (Stevens et al., 2007, Stephan et al., 2012, Hong et al., 2016, Kodavali et al., 2014, Prasad et al., 2016). The demonstration that functional allelic variation at the *C4* gene explains much of the well-known genetic association between the MHC locus and schizophrenia further supports a role in the disorder for the classical pathway of complement (Sekar et al., 2016). Taken together, these data support a scenario where dysregulated activation of the complement classical pathway is, at least in part, responsible for the reduced synapse number that typifies the brain in schizophrenia. Much remains to be discovered, notably when during disease development these events occur, and therefore identifying complement system changes in the FEP population is particularly important.

The few published studies seeking biomarker evidence of complement activation and dysregulation in adults with schizophrenia are confusing and contradictory (Mayilyan et al., 2008, Hakobyan et al., 2005, Wong et al., 1996). As a first step towards clarification, we measured selected complement markers in serum from FEP and controls in samples from the PUMP and GAP studies. Prior to consideration of independently contributing variables, one complement marker emerged as significantly different between the groups. TCC, a marker of terminal pathway activation, was significantly lower in serum of FEP patients compared to controls. Measured TCC values in all samples were higher than expected, in part because only archived serum samples were available – complement activation markers are best measured in freshly obtained EDTA plasma to minimise artefactual ex vivo activation. The key contributing independent variables considered were age, sex, ethnicity, tobacco and cannabis smoking. A significant impact of tobacco smoking on biomarker measurements was previously described in other studies (Kew et al., 1985, Robbins et al., 1991); smoking has been shown to cause complement activation and induce an acute phase response likely to impact levels of complement proteins (Kobayashi et al., 1988, Sunyer et al., 2009). A recent meta-analysis also established that daily tobacco use is associated with an increased risk of psychotic disorder and an earlier age at onset of psychotic illness (Gurillo et al., 2015). A notable interdependence was seen between TCC levels and tobacco smoking; TCC levels were lower both in FEP cases and smokers and the significance of TCC for differentiating cases and controls was lost when smoking was corrected for.

The multivariate models showed interdependence between complement analytes and demographic and lifestyle variables. The chosen model (Model B) revealed, after stepwise selection, two complement proteins, C3 and C4, as significantly different between the FEP and control groups, with four complement analytes (C5, TCC, C1 inhibitor and FHR125) trending towards significance and strengthening the model. ROC analysis demonstrated a predictive value (AUC) for the model of 0.79 for distinguishing FEP from controls, considered “moderately predictive” (Greiner et al., 2000). Complement analytes alone or demographics (sex, age, ethnicity) contributed to similar degrees to the predictive value (AUC 0.63 and 0.69 respectively). It is notable that the complement analytes selected in the model have roles in the classical pathway. C4 is an essential classical pathway component, implicated by genetic and expression studies in the pathology of schizophrenia, C3 is the key complement protein essential for efficient opsonisation regardless of the initiating pathway; while C1inh is the sole plasma regulator of classical pathway activation. The findings therefore support a role for classical pathway dysregulation in the early stages of development of schizophrenia. Interestingly, C3 and C4 levels are also associated with metabolic syndrome development, which is commonly associated with established psychosis (Liu et al., 2016). Despite the described complement dysregulation suggesting inflammatory component in FEP pathogenesis, there is no significant alteration of CRP. The disconnection between inflammation and CRP levels is not without precedent and has been reported in several other inflammatory conditions, including SLE (Gaitonde et al., 2008).

The current data set has several limitations: the sample size is small, the patient group heterogeneous and biased in terms of sex and ethnicity. The serum samples available for testing are not ideal for measurement of complement analytes which are prone to ex vivo artefact. A further limitation is the fact that the majority of the patients were not drug naïve at the time of blood sample collection and details of drug therapy were not available. There is a possibility that antipsychotic treatment drives some of these differences, as described for cytokines in the past (Baumeister et al., 2016). In our study, we cannot control entirely for the effect of each drug individually. The immediate previous history of infectious illness or anti-inflammatory drug history was also not available for the analysis. Groups more representative of the general population (matched for condition, but also smoking habit and ethnicity) would help to reduce the number of incorrectly classified cases, in particular the number of false positives.

Our findings suggest that measurement of selected complement analytes interrogating the classical pathway could contribute to the early diagnosis of psychosis. A large study, planned prospectively, minimising sample bias and using appropriately collected and stored EDTA plasma samples, is needed to confirm and extend the findings. It is also important to explore complement biomarkers in individuals considered at high risk of developing schizophrenia and patients with established schizophrenia to discover whether complement dysregulation is a feature of very early disease and if it is maintained in later disease stages.

The following are the supplementary data related to this article.

Supplementary tables Image 1

Supplementary Fig. 1 Spearman's correlation coefficients between analytes. Only the significant correlations at p = 0.05 are provided, a blank cell representing no significance. Some mild but no strong positive correlations can be observed, with a maximum coefficient of 0.44 between C1inh and FB. Supplementary Fig. 1

## Contributors

MK and WZ performed all the laboratory analyses; ST performed all statistical analyses and developed the models used; FG, MDF, VM and RM collected, archived and supplied patient/control samples and data; MOD provided critical expertise in planning the study; MK wrote the first draft of the manuscript; BPM conceived and planned the study and oversaw the data handling and manuscript preparation; all authors contributed to and have approved the final manuscript.

## Conflict of interest

FG has received support or honoraria for CME, advisory work and lectures from Bristol- Myers Squibb, Janssen, Lundbeck, Otsuka, Roche, and Sunovion, has research funded by an NHS Innovations/Janssen-Cilag award and has a family member with professional links to Lilly and GSK, including shares. FG is in part funded by the National Institute for Health Research Collaboration for Leadership in Applied Health Research & Care Funding scheme and by the Stanley Medical Research Institute. VM received honoraria from Lundbeck and research funding from Johnson and Johnson not relevant for this project. MDF received honoraria from Lundbeck. RMM received honoraria from Janssen, Lundbeck, Otsuka, Lilly, and Sunovian. BPM is a Consultant for GSK. Cardiff University has received an honorarium from Roche for a presentation by MOD in 2015.
