# Supplementary material for: Complement system biomarkers in first episode psychosis
Source: Schizophr Res. 2019 Feb;204:16–22. doi: 10.1016/j.schres.2017.12.012 (PMC6406022; doi:10.1016/j.schres.2017.12.012)
Supplement: Supplementary file 1 — Supplementary tables [file mmc1.docx]

**A**

| **Step** | **Variable removed** | **AIC** |
| --- | --- | --- |
| 0: full model | none | 192.56 |
| 1 | C1q | 190.56 |
| 2 | FHR125 | 188.56 |
| 3 | Properdin | 186.57 |
| 5 | C4 | 184.65 |
| 6 | CR1 | 182.98 |
| 7 | C1Inh | 181.62 |
| 8 | FB | 180.41 |
| 9 | FH | 179.3 |
| 10 | C3 | 178.47 |
| 11 | C5 | 178.23 |

**B**

| **Step** | **Variable removed** | **AIC** |
| --- | --- | --- |
| 0: full model | none | 114.45 |
| 1 | Properdin | 112.5 |
| 2 | Cannabis | 110.78 |
| 3 | Age | 109.18 |
| 5 | C1q | 108.6 |
| 6 | FB | 107.33 |
| 7 | CRP | 107.17 |
| 8 | FH | 107.08 |

Supplementary Table 1. Analysis showing which variables were removed at each step of the selection process in each model (A – Model A; B – Model B), as well as the respective AIC statistic.

| **Assay** | **Tobacco smokers** | | **Tobacco non-smokers** | | **p-value** |
| --- | --- | --- | --- | --- | --- |
|  | **Mean** | **StDev** | **Mean** | **StDev** |  |
| C1q | 127.96 | 27.34 | 134.33 | 27.34 | 0.15 |
| C3 | 1931.46 | 2461.63 | 1276.68 | 2461.63 | 0.34 |
| C4 | 484.71 | 131.65 | 452.07 | 131.65 | 0.15 |
| C5 | 80.29 | 37.36 | 94.02 | 37.36 | 0.41 |
| FB | 106.42 | 36.81 | 106.02 | 36.81 | 0.66 |
| FH | 401.66 | 185.08 | 328.85 | 185.08 | **0.05** |
| C1inh | 105.33 | 34.26 | 110.32 | 34.26 | 0.61 |
| Properdin | 4.27 | 2.72 | 4.04 | 2.72 | 0.84 |
| FHR125 | 9.88 | 6.41 | 10.70 | 6.41 | 0.52 |
| TCC | 30.03 | 14.06 | 36.80 | 14.06 | **0.01** |
| CR1 | 0.02 | 0.01 | 0.02 | 0.01 | 0.82 |
| CRP | 5.25 | 5.94 | 3.21 | 5.94 | 0.10 |

Supplementary Table 2. Influence of tobacco smoking on complement analytes. Significance of differences was tested using the Mann-Whitney test. Variables significant at p = 0.05 are in bold and underlined. TCC; terminal complement complex; C1inh; C1 inhibitor; FB; Factor B; FH; Factor H; FHR125; Factor H related proteins 1, 2, and 5; CR1; complement receptor 1; CRP; C-reactive protein.

| **Assay** | **Cannabis smokers** | | **Cannabis non-smokers** | | **p-value** |
| --- | --- | --- | --- | --- | --- |
|  | **Mean** | **StDev** | **Mean** | **StDev** |  |
| C1q | 127.21 | 28.06 | 134.83 | 28.06 | 0.052 |
| C3 | 1735.84 | 2216.09 | 1911.84 | 2216.09 | 0.23 |
| C4 | 464.27 | 120.82 | 510.59 | 120.82 | 0.09 |
| C5 | 76.57 | 38.11 | 100.35 | 38.11 | **0.002** |
| FB | 105.80 | 39.29 | 107.69 | 39.29 | 0.48 |
| FH | 397.16 | 183.12 | 356.14 | 183.12 | 0.21 |
| C1inh | 106.27 | 34.69 | 106.88 | 34.69 | 0.86 |
| Properdin | 4.42 | 2.65 | 3.72 | 2.65 | 0.14 |
| FHR125 | 9.84 | 6.23 | 10.63 | 6.23 | 0.54 |
| TCC | 31.87 | 14.65 | 30.68 | 14.65 | 0.77 |
| CR1 | 0.02 | 0.00 | 0.03 | 0.00 | 0.08 |
| CRP | 4.78 | 5.66 | 4.84 | 5.66 | 0.64 |

Supplementary Table 3. Influence of cannabis smoking on complement analytes. Significance of differences was tested using the Mann-Whitney test. Variables significant at p = 0.05 are in bold and underlined. TCC; terminal complement complex; C1inh; C1 inhibitor; FB; Factor B; FH; Factor H; FHR125; Factor H related proteins 1, 2, and 5; CR1; complement receptor 1; CRP; C-reactive protein.

| **Assay** | **Male** | | **Female** | | **p-value** |
| --- | --- | --- | --- | --- | --- |
|  | **Mean** | **StDev** | **Mean** | **StDev** |  |
| C1q | 129.68 | 27.15 | 130.06 | 30.52 | 0.80 |
| C3 | 1709.80 | 1991.75 | 1793.86 | 2041.21 | 0.78 |
| C4 | 487.28 | 123.21 | 445.71 | 145.07 | **0.047** |
| C5 | 81.15 | 39.64 | 88.55 | 41.54 | 0.14 |
| FB | 101.90 | 37.02 | 106.65 | 36.71 | 0.36 |
| FH | 383.99 | 176.49 | 364.84 | 173.25 | 0.49 |
| C1inh | 102.84 | 35.76 | 102.12 | 28.39 | 0.91 |
| Properdin | 4.35 | 2.67 | 4.40 | 3.06 | 0.96 |
| FHR125 | 9.69 | 6.81 | 10.35 | 4.67 | 0.21 |
| TCC | 30.23 | 13.51 | 34.36 | 15.52 | **0.04** |
| CR1 | 0.02 | 0.01 | 0.02 | 0.01 | 0.65 |
| CRP | 4.84 | 5.59 | 3.97 | 4.44 | 0.37 |

Supplementary Table 4. Influence of sex variable on complement analytes. Significance of differences was tested using the Mann-Whitney test. Variables significant at p = 0.05 are in bold and underlined. TCC; terminal complement complex; C1inh; C1 inhibitor; FB; Factor B; FH; Factor H; FHR125; Factor H related proteins 1, 2, and 5; CR1; complement receptor 1; CRP; C-reactive protein.
