# Supplementary material for: Complement system biomarkers in first episode psychosis
Source: Schizophr Res. 2019 Feb;204:16–22. doi: 10.1016/j.schres.2017.12.012 (PMC6406022; doi:10.1016/j.schres.2017.12.012)
Supplement: Supplementary Fig. 1 — Spearman's correlation coefficients between analytes. Only the significant correlations at p = 0.05 are provided, a blank cell representing no significance. Some mild but no strong positive correlations can be observed, with a maximum coefficient of 0.44 between C1inh and FB. [file mmc2.docx]

**Supplementary Data**


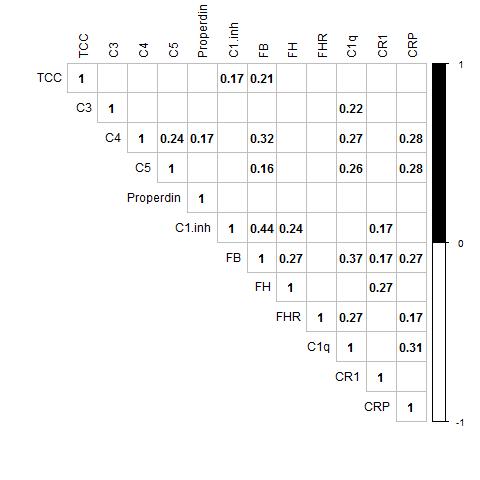


Supplementary Figure 1. Spearman’s correlation coefficients between analytes. Only the significant correlations at p= 0.05 are provided, a blank cell representing no significance. Some mild but no strong positive correlations can be observed, with a maximum coefficient of 0.44 between C1inh and FB.
